# Comparison of Mini-FLOTAC, Flukefinder^®^ and sedimentation techniques for detection and quantification of *Fasciola hepatica* and *Calicophoron daubneyi* eggs using spiked and naturally infected bovine faecal samples

**DOI:** 10.1186/s13071-023-05890-2

**Published:** 2023-08-02

**Authors:** Antonio Bosco, Lavinia Ciuca, Maria Paola Maurelli, Paola Vitiello, Giuseppe Cringoli, Joaquin M. Prada, Laura Rinaldi

**Affiliations:** 1 Department of Veterinary Medicine and Animal Production, University of Naples Federico II, Regional Center for Monitoring Parasitic infections (CREMOPAR), Naples, Italy; 2 School of Veterinary Medicine, Faculty of Health and Medical Sciences, University of Surrey, Guilford, UK

**Keywords:** *Fasciola hepatica*, *Calicophoron daubneyi*, Ruminants, Mini-FLOTAC, Flukefinder^®^, Sedimentation, Latent class analysis

## Abstract

**Background:**

Fasciolosis (*Fasciola hepatica*) and paramphistomosis (*Calicophoron daubneyi*) are two important infections of livestock. *Calicophoron daubneyi* is the predominant Paramphistomidae species in Europe, and its prevalence has increased in the last 10–15 years. In Italy, evidence suggests that the prevalence of *F. hepatica* in ruminants is low in the southern part, but *C. daubneyi* has been recently reported at high prevalence in the same area. Given the importance of reliable tools for liver and rumen fluke diagnosis in ruminants, this study evaluated the diagnostic performance of the Mini-FLOTAC (MF), Flukefinder^®^ (FF) and sedimentation (SED) techniques to detect and quantify *F. hepatica* and *C. daubneyi* eggs using spiked and naturally infected cattle faecal samples.

**Methods:**

Briefly, negative bovine faecal samples were artificially spiked with either *F. hepatica* or *C. daubneyi* eggs to achieve different egg count levels: 10, 50 and 100 eggs per gram (EPG) of faeces. Moreover, ten naturally infected cattle farms from southern Italy with either *F. hepatica* and/or* C. daubneyi* were selected. For each farm, the samples were analysed individually only with MF technique and as pools using MF, FF and SED techniques. Bayesian latent class analysis (LCA) was used to estimate sensitivity and accuracy of the predicted intensity of infection as well as the infection rate in the naturally infected farms.

**Results:**

The outcome of this study showed that the highest number of eggs (*F. hepatica* and *C. daubneyi*) recovered was obtained with MF, followed by FF and SED in spiked infected samples at 50 and 100 EPG, while at lower infection levels of 10 EPG, FF gave the best results. Moreover, the sensitivity for all the techniques included in the study was estimated at > 90% at infection levels > 20 EPG for both *F. hepatica* and *C. daubneyi* eggs. However, MF was the most accurate of the three techniques evaluated to estimate fluke infection intensity. Nevertheless, all three techniques can potentially estimate infection rate at farm level accurately.

**Conclusions:**

Optimization and standardization of techniques are needed to improve the FEC of fluke eggs.

**Graphical Abstract:**

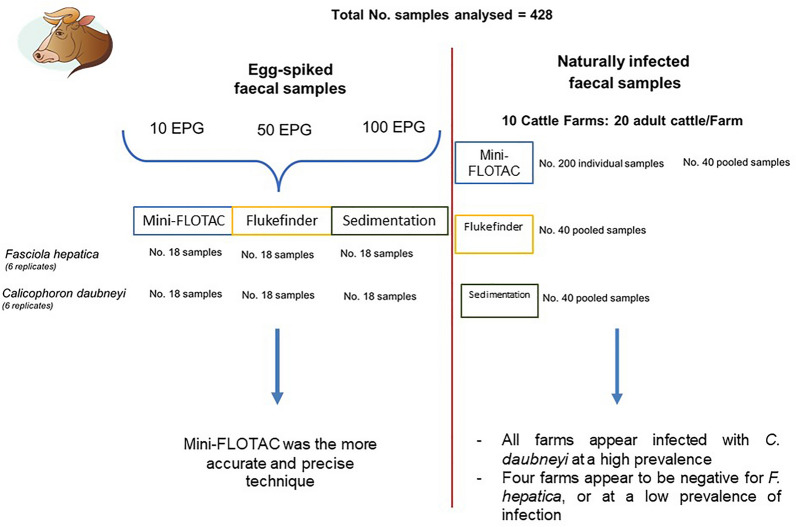

## Background

Among the parasitic helminths that freshwater snails (e.g. *Galba truncatula*) can transmit, *Fasciola hepatica* (liver fluke) and *Calicophoron daubneyi* (rumen fluke) are wide distributed in temperate countries. Fasciolosis is highly endemic mainly in Western Europe [1–4], causing losses of production with significant costs at > $3 billion per year for the global livestock farming business [5].

Paramphistomosis is considered an emerging parasitic disease of ruminants in Europe [6]. Over the last 20 years, its incidence and prevalence have increased significantly in Europe and many outbreaks of clinical manifestation have been reported in different countries mainly induced by *C. daubneyi*, the predominant Paramphistomidae species in Europe [7–11]. Clinical paramphistomosis is mostly caused by a large burden of juvenile flukes in the small intestine of the infected host, as adult parasites, generally located in rumen and reticulum, are quite well tolerated [12].

In Italy the prevalence of *F. hepatica* in ruminants appears to be low (0.7–6.0% in sheep and 0.9–7.8% in cattle); however *C. daubneyi* has been recently reported at high prevalence (4.5–51.1% in sheep and 9.6–60.9% in cattle) in the same area [13–15].

Diagnosis is very important in performing the most effective programmes for fasciolosis and paramphistomosis control [16].

The results of a survey by Hoyle et al. [17] revealed confusion amongst sheep and/or cattle farmers over the diagnosis and control of flukes, highlighting the need to provide best practice advice. Diagnosis and monitoring of fasciolosis and paramphistomosis in ruminants are challenging. Usually, liver fluke presence information on a farm occurs from liver condemnation reports [18], which are based on visual inspections at abattoirs, but these procedures are variable and potentially erroneous, because they are not standardized [19]. Several tests for ante-mortem diagnosis of flukes exist, but none can be considered sufficiently sensitive and specific for use in the field [20] as ‘pen-side test’. Coproantigen-based techniques are promising tools in fluke diagnosis; they showed 100% sensitive to detect experimental ovine fasciolosis and give information about correlation between fluke burden and coproantigen amounts [21]. However, ELISA-based techniques cannot be used directly on farm and have not been validated extensively under field conditions to confirm the sensitivity obtained in experimental infections [22].

Fluke faecal egg counts (flukeFECs) are, instead, simple and rapid [19] and can also be performed directly on farm, because neither specialist sampling techniques nor sophisticated laboratory equipment is required. FlukeFECs are routinely used in veterinary parasitic diagnosis, with almost 100% specificity, although they are able to detect only patent infections [20]. There is no gold-standard diagnostic tool for fluke infection so often a combination of clinical signs, grazing history, serological, coproantigen and flukeFECs and/or abattoir reports are used to confirm fluke infections. Several variations of flukeFECs have been developed, using simple sedimentation (SED) [23], sedimentation combined with flotation [23], sedimentation with fine filtration [24], Flukefinder^®^ technique (FF) [25] or flotation with (Mini-) FLOTAC techniques [1, 26]. Given the importance of reliable tools for liver and rumen fluke diagnosis in ruminants, this study aimed to evaluate the diagnostic performance of the MF, FF and SED techniques to detect and quantify *F. hepatica* and *C. daubneyi* eggs using spiked and naturally infected cattle faecal samples.

## Methods

### Egg-spiked faecal samples

Negative bovine faecal samples were artificially spiked with either *F. hepatica* or *C. daubneyi* eggs to achieve different egg count levels of 10, 50 or 100 eggs per gram (EPG) of faeces. The *F. hepatica* and *C. daubneyi* positive and negative faecal samples were collected from adult cattle (> 24 months old) in three farms located in the Campania region (southern Italy). Naturally infected positive samples by one of the two flukes were collected from grazing cattle, whilst negative samples were collected from housed dairy cattle without pasture access.

**Fig. 1:**
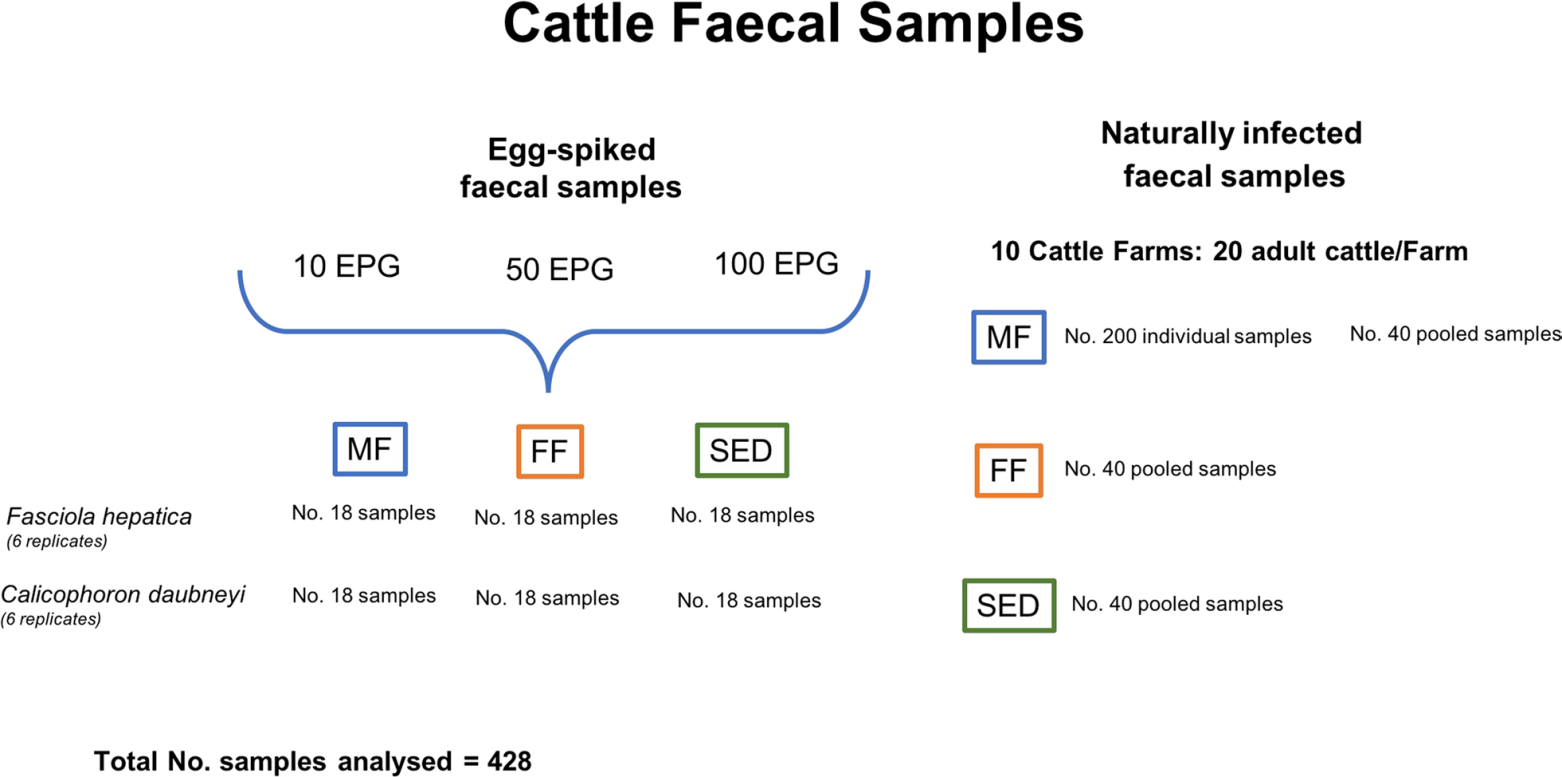
Number of egg-spiked and naturally infected (individually or pooled) faecal samples analysed using Mini-FLOTAC (MF), Flukefinder^®^ (FF) and sedimentation (SED) techniques for detection and quantification of *Fasciola hepatica* and *Calicophoron daubneyi* eggs

To identify the positive samples for potential egg extraction, as well as the negative samples to be experimentally infected, each sample was analysed in five replicates by the FLOTAC basic technique (sensitivity = 94% and specificity = 98%) with a detection limit of 1 EPG of faeces using zinc sulphate flotation solution (specific gravity = 1.35) [14, 27]. The positive cattle were used as donors for the extraction of *F. hepatica* and *C. daubneyi* eggs from faeces, using the egg recovery technique described by Bosco et al. [28] with some modifications. Briefly, four sieves of different mesh size (1 mm, 250 μm, 212 μm and 63 μm) were employed to separate the fluke eggs from the faeces. The 63-μm sieve was washed with tap water to recover eggs and sedimented in a conical beaker for 4 min. The supernatant was eliminated, and the sediment obtained was a purified suspension of eggs. The extraction method was used separately for the two flukes to obtain mono-infected samples. The purified eggs obtained for each fluke were suspended in distilled water to determine their concentration by calculating the arithmetic mean of egg counts in ten aliquots of 10 μl each. Three concentrations of 10, 50 and 100 EPG were prepared, adding appropriate egg suspensions to three negative faecal samples (helminth free) and homogenizing them. Six replicates of each sample were analysed using the three methods (MF, FF and SED) (Fig. 1).

### Naturally infected faecal samples

Ten farms with fluke positive cattle (infections with *C. daubneyi* and/or *F. hepatica*) in southern Italy were selected. These farms were identified from the diagnostic activity of the Regional Center for Monitoring Parasitic infections (CREMOPAR, Campania region, southern Italy).

In each farm, individual faecal samples were collected directly from the rectum of 20 adult cattle (> 12 months). For each farm, the samples were analysed individually only with MF technique and as pools using also FF and SED techniques. The four pools of faeces (each consisting of 5 samples) were performed using the protocol described by Rinaldi et al. [29] (Fig. 1).

The three copromicroscopic methods were performed for both the spiked and the naturally infected samples following the manufacturer’s instructions. The faecal egg counts (FECs; expressed in EPG) were obtained using a multiplication factor of 5 for MF (0.2 g of faeces examined = 2 ml of faecal suspension which contains 5 g in a total volume 50 ml), 0.5 for FF (2 g of faeces examined) and 0.1 for SED (10 g of faeces examined) (Table 1).

**Table 1:** Schematic features of Mini-FLOTAC (MF), Flukefinder^®^ (FF) and sedimentation (SED) techniques included in the study

FEC techniques	Amount of faeces used (grams)	Quantity of faeces examined (grams)	Detection limit (EPG) and multiplication factor
MF	5	0.2	5
FF	2	2	0.5
SED	10	10	0.1

###  Statistical analyses

The sensitivity of the three techniques as well as the accuracy in predicting the intensity of infection was estimated from the egg-spiked faecal samples. First, the percentage recovery of fluke eggs was calculated to assess the accuracy of FEC for each technique at each level of egg count, using the following formula: % egg recovery = 100 – (true FEC – observed FEC)/true FEC × 100 [28]. Moreover, a simple model was developed to estimate the overdispersion of the eggs counted with the different techniques, assuming measurement error is distributed according to a negative binomial (such as in Prada et al. [30]), which is a more flexible assumption than using a Poisson, like in Atljia et al. [31]. As the size of the faecal sample examined (in grams) is different across the three techniques, the dosage in EPG needs to be transformed to the number of eggs expected in the faecal sample (Table 1). The model is run in a Bayesian framework using a *Gibbs* sampling package in R [32], “jags” [33] and “runjags” [34], with a burn in of 1000 (discarded runs), drawing a total of 10,000 samples with a thinning of 10.

Preliminary simulations (not shown) suggested that overdispersion was the same across the two parasite species examined; thus, a single parameter was used for each diagnostic technique. Using the posterior distributions of overdispersion obtained from the model above, we simulated 1000 repeated measurements across a range of true intensity of infection measured in EPG from 1 to 100 (at 0.5 EPG steps, 199,000 total samples) for the three diagnostics. We then calculated the sensitivity (proportion of samples correctly identified as positive) and accuracy in estimating intensity of infection by comparing the true intensity of infection to those estimated through the different diagnostics.

To estimate the infection rate in the farms naturally infected, we developed a Bayesian latent class analysis (LCA) model, following recent work in other parasites [30]. The infection status of each individual animal is estimated from the different diagnostics (both individual-level MF and their contribution to the pooled samples). Each infected individual will have their intensity of infection (true egg count) drawn from a gamma distribution. The parameters for the gamma distribution for *C. daubneyi* are estimated by the model; however, for *F. hepatica*, due to the low number of positive samples, it could not be estimated from these data, so it was calculated from the data reported in Rinaldi et al. [1]. The number of eggs in each pool is assumed to be the average of the true number of eggs across the five individuals contributing to that pool. The data from the different diagnostics are assumed to be generated from a negative binomial draw of the true number of eggs (in the individual or the pool). The overdispersion value needed was drawn from the posterior distributions generated from the model above for the egg-spiked faecal samples. Farm-level infection rate can then be estimated, and the expected farm infection rate with FF and SED, which were not collected at the individual level, can be simulated. As before, we used the “jags” [33] and “runjags” [34] packages to run the model; we again discarded 1000 runs (burn in), drawing a total of 10,000 samples without thinning. All code is available at: https://github.com/joaquinprada/Fluke-MF-FF-SED-Comparison.

##  Results

**Fig. 2:**
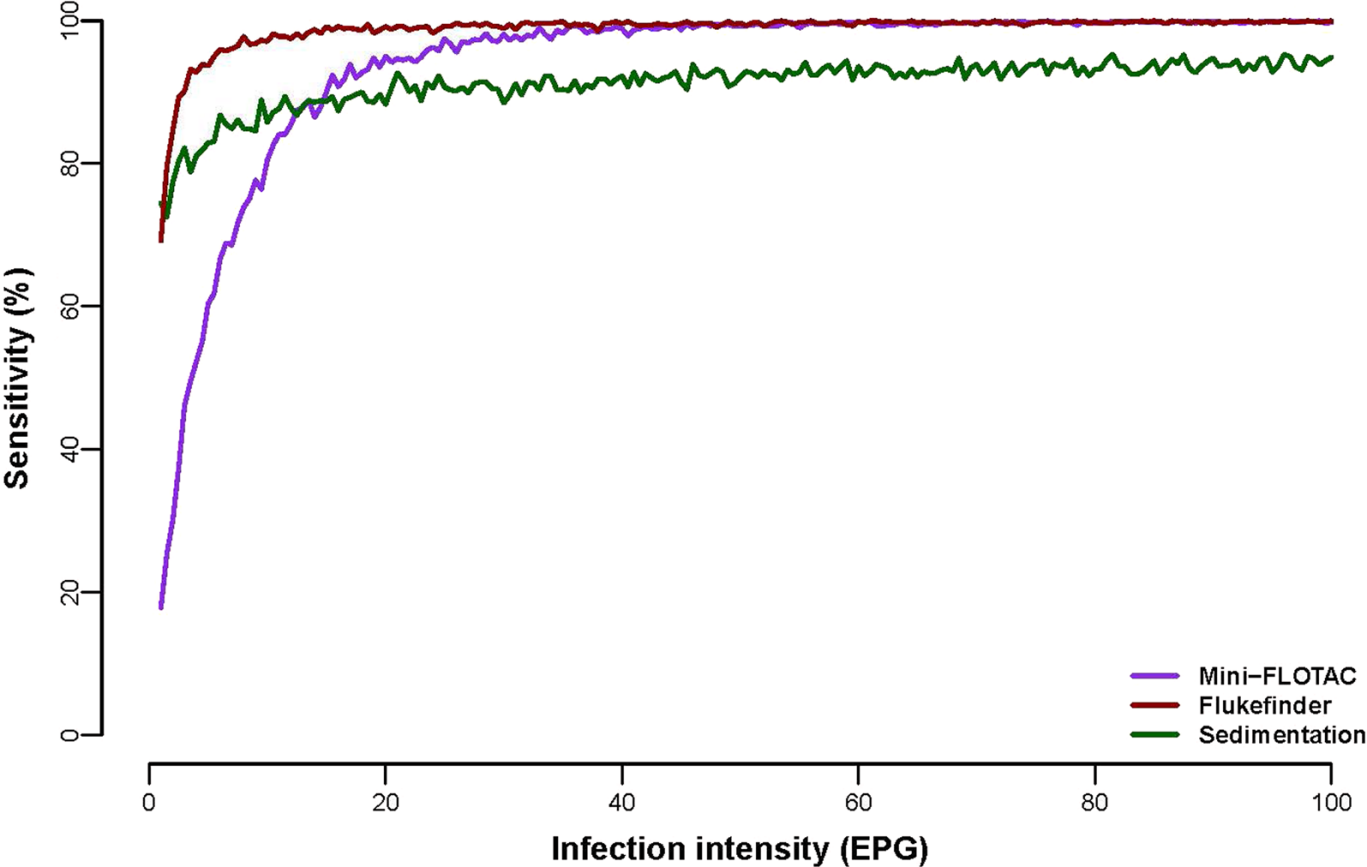
Estimated sensitivity of the three diagnostic techniques, Mini-FLOTAC (MF), Flukefinder^®^ (FF) and sedimentation (SED), across a range of infection intensities, measured in eggs per gram (EPG) of faeces

**Fig. 3:**
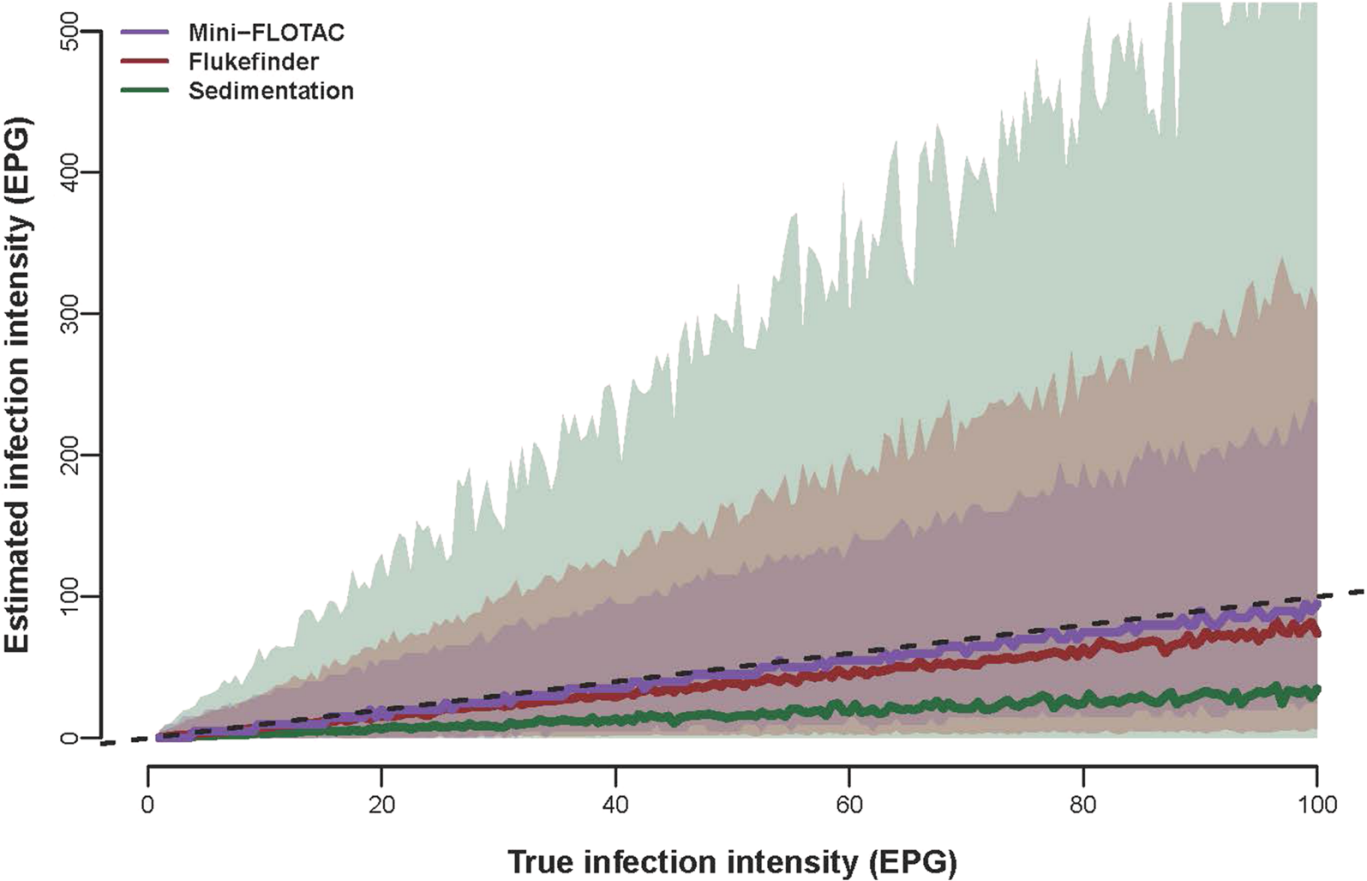
Comparison between true and estimated infection intensity in eggs per gram (EPG) of faeces. Dashed diagonal black line indicates where true and estimated EPGs are equal. The median estimated infection intensity is represented by the solid lines, while the shaded area shows the 95% credible interval, across the three diagnostic techniques, Mini-FLOTAC (MF), Flukefinder^®^ (FF) and sedimentation (SED)

Table 2 reports the outcome of the EPG levels of *F. hepatica* and *C. daubneyi* using egg-spiked cattle faecal samples at different known EPG concentrations (10, 50, 100). The results were expressed as mean EPG of the six replicates comparing the performances of three different techniques included in the study (MF, FF and SED). The egg-spiking test revealed that all the methods were able to recover eggs of *F. hepatica* and *C. daubneyi* from cattle faeces, with MF having the lowest sensitivity below 15 EPG, and SED having the lowest above 15 EPG (Fig. 2). Nevertheless, all three techniques had a sensitivity > 90% at infection levels above 20 EPG. Moreover, when comparing true and estimated intensity of infection (in EPG) with the different diagnostics, MF was the technique that provided the better estimation, in terms of both accuracy (median estimated EPG) and precision (size of the 95% credible interval) (Fig. 3).

**Table 2:** Mean number of detection and percentage recovery of *Fasciola hepatica* (*FH*) and *Calicophoron daubneyi* (*CD*) eggs at the different egg count levels using spiked infected cattle faecal samples performed with Mini-FLOTAC (MF), Flukefinder^®^ (FF) and sedimentation (SED) techniques included in the study

Fluke	FEC techniques	10 EPG		50 EPG		100 EPG	
		Detected EPG	% egg recovery	Detected EPG	% egg recovery	Detected EPG	% egg recovery
*FH*	MF	2.5	25.0	25.8	51.6	64.2	64.2
	FF	3.2	32.0	13.0	26.0	48.8	48.8
	SED	0.7	7.0	1.5	3.0	10.4	10.4
*CD*	MF	1.7	17.0	29.2	58.4	70.8	70.8
	FF	3.8	38.0	15.5	31.0	52.2	52.2
	SED	1.1	11.0	1.7	3.4	11.6	11.6

*EPG* eggs per gram of faeces

**Fig. 4:**
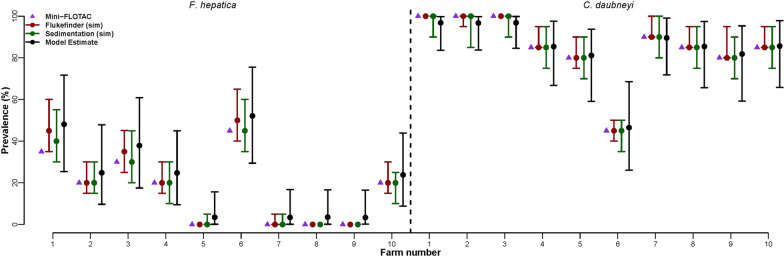
Infection rate of *Fasciola hepatica* (left) and *Calicophoron daubneyi* (right) across ten farms in Southern Italy. Infection rate by Mini-FLOTAC was calculated from the individual-level samples (purple), while median infection rate by Flukefinder^®^ (dark red) and sedimentation (dark green) was estimated from the simulated individual-level data. The model estimate, considering all three diagnostic techniques, and both individual-level and pooled samples, is shown in black. Error bars show the 95% credible interval

When evaluating natural infection across ten farms in southern Italy, while all farms appear infected with *C. daubneyi* at a high infection rate, four appear to be negative for *F. hepatica* or at a low infection rate (Fig. 4). Overall, the infection rate estimated by the individual-level MF technique is very similar to the model estimated infection rate, albeit potentially underestimating infection rate when infection is low (Fig. 4—left side). Simulated individual-level FF and SED diagnostic results also yield comparable infection rate values across all farms for both parasite species (Fig. 4—dark red and dark green). The mean EPGs across the pooled samples were higher for MF and FF compared to SED (Table 3).

**Table 3:** Eggs per gram (EPG) of faeces mean of *Fasciola hepatica* (*FH*) and *Calicophoron daubneyi* (*CD*) using naturally infected bovine faecal samples from ten farms analysed individually (no. 20 samples for each farm) only with Mini-FLOTAC (MF) technique and as pooled faecal samples (no. 4 pools samples for each farm) using also Flukefinder^®^ (FF) and sedimentation (SED) techniques included in the study

No. cattle farm	MF				FF		SED	
	Individual faecal samples (EPG mean)		Pooled faecal samples (EPG mean)		Pooled faecal samples (EPG mean)		Pooled faecal samples (EPG mean)	
	*FH*	*CD*	*FH*	*CD*	*FH*	*CD*	*FH*	*CD*
1	2.50	151.50	2.50	147.50	0.73	71.33	3.88	126.25
2	1	68.5	1.25	65	0.38	32.88	3.00	52.63
3	1.50	153.25	1.25	148.75	0.40	67.25	3.13	131.38
4	1	45	1.25	40	0.13	18.70	2.38	34.88
5	0	25.25	0	22.50	0	7.25	0	20.38
6	2.25	18.25	2.50	16.25	0.58	7.98	3.38	14.88
7	0	142.75	0	135	0	45.88	0	32.63
8	0	53	0	45	0	16.13	0	32.63
9	0	171	0	168.75	0	50.55	0	132
10	1	67	1.25	65	0.2	30.63	2.13	54.88

##  Discussion

In this study MF was compared for the first time to our knowledge with FF and SED for detection of liver and rumen flukes in spiked and naturally infected cattle faecal samples. The MF was already successfully used by Malrait et al. [26] to assess the presence of rumen fluke infections and for FEC of *C. daubneyi* eggs, showing that it was a reliable method, with sensitivity and specificity of 94% and 98%, respectively. In our study, the MF technique permitted the recovery of the highest number of eggs, followed by FF and SED for spiked infected samples at 50 and 100 EPG, while lower infection level of 10 EPG FF gave the best results. These abovementioned findings were similar for both flukes. In particular, the highest percentages of recovery were obtained with MF at 100 EPG, but the retrieved values—64.2% for liver flukes and 70.8% for rumen fluke—were lower than those reported in previous studies for gastrointestinal nematodes (GIN) in cattle (98.1% by Amadesi et al. [35]; 70.9% by Paras et al. [36]), sheep (100% by Bosco et al. [28] and Godber et al. [37]) and horses (74.2% by Napravnikova et al. [38]), but higher than equine strongyle eggs reported by Noel et al. [39] (42.6%). This difference can be explained by the fact that fluke eggs are heavy, while GINs of ruminants and horses are light eggs; therefore, the behaviour in flotation solutions and the recovery rate are completely different [27, 28, 35, 40]. However, the SED was the least efficient technique, recovering the lower rate of eggs. Our results agree with a previous study on comparison between FF and SED techniques showing that FF was more efficient than simple Becker sedimentation to retrieve *F. hepatica* eggs in sheep and cattle faeces; in fact, it was able to detect 2 EPG with a higher sensitivity (100%) already at low levels of infection [19]. Although the sedimentation methods (i.e. simple, with fine filtration, followed by flotation) are the most used for fluke infection detection, their sensitivity is low [16, 19]. The estimated sensitivity for all the techniques included in the study was > 90% when the intensity of infection was > 20 EPG (Fig. 2). However, at very low intensities of infection, sensitivity was estimated to drop, particularly for MF, which has a detection limit of 5 EPG. The findings obtained with MF agree with Zarate-Rendon et al. [41], showing that MF and FF gave better results than Kato-Katz for FEC of *F. hepatica* also in humans, with a sensitivity of 100% at 96 EPG level, but the sensitivity decreased at 40% for MF and at 60% for FF at level of 14 EPG. In our previous studies in which MF and FLOTAC were compared for the detection of fluke eggs, the outcome revealed that FLOTAC had a higher sensitivity than MF at low levels of infection. The step of centrifugation in the FLOTAC technique, which is missing in the MF method, helped to increase the number of fluke eggs detected [40, 42].

On the other hand, MF is the most accurate of the three techniques to estimate the intensity of infection, which aligns with previous work on GIN in ruminants and horses [28, 35, 43]. FF and SED however can over- and under- estimate intensity of infection more, with these results consistent across the whole infection intensity range evaluated (from 1 to 100 EPG). In other studies [35, 44, 45], the accuracy improves when the EPG in faecal sample increases. However, it is very important to use diagnostic tools with a low detection limit and high accuracy also at low levels of infection, because also the presence of only few flukes (especially for *F. hepatica* and sheep, which are highly sensitive to this parasite) can cause significantly reduced productivity [46, 47].

Findings obtained by MF from naturally infected cattle showed that individual and pooled samples did not give statistically different results for all ten farms analysed for both flukes, as previously shown also for GINs in ruminants [29, 42, 48–50]. Moreover, the estimated prevalence with the model, which includes the information across the three techniques, aligns with the infection rate measured with MF and the infection rate estimated from the simulated FF and SED techniques. All farms showed presence of *C. daubneyi* at very high infection rate, while *F. hepatica* was more moderate in most farms, four of which had no positive samples recovered, which would suggest no infection (or at a relatively low infection rate). Due to the low number of *F. hepatica* eggs detected, further studies are needed, as it was not possible to assess the intensity of infection in the region.

## Conclusion

Optimization and standardization of techniques are needed to improve the FEC of fluke eggs. The combination of sensitive, accurate, precise and standardized FEC techniques with a reliable automated system will permit not only an efficient observation, but also a quantification of parasitic elements thanks to the use of an artificial intelligence software [51, 52]. MF is an accurate technique to estimate intensity of infection at moderate to high levels and can be recommended in endemic areas, for example for diagnosing *C. daubneyi* in southern Italy.

Finally, further studies could be important to support informed treatment decisions as well as to determine the threshold for ‘economically relevant’ infection levels and to evaluate the diagnostic performance of the different tests around this threshold.

## Data Availability

All data generated or analysed during this study are included in this published article. The datasets used and/or analysed during the present study available from the corresponding author upon reasonable request.

## Abbreviations

MF: Mini-FLOTAC
FF: Flukefinder^®^
SED: Sedimentation
EPG: Eggs per gram
FEC: Faecal egg count
